# Calcium Signaling in Live Cells on Elastic Gels under Mechanical Vibration at Subcellular Levels

**DOI:** 10.1371/journal.pone.0026181

**Published:** 2011-10-28

**Authors:** Wagner Shin Nishitani, Taher A. Saif, Yingxiao Wang

**Affiliations:** 1 Department of Bioengineering and Beckman Institute for Advanced Science and Technology, University of Illinois, Urbana-Champaign, Urbana, Illinois, United States of America; 2 Department of Mechanical Science and Engineering, University of Illinois, Urbana-Champaign, Urbana, Illinois, United States of America; 3 Integrative and Molecular Physiology, Center for Biophysics and Computational Biology, Institute for Genomic Biology, University of Illinois, Urbana-Champaign, Urbana, Illinois, United States of America; 4 The Capes Foundation, Ministry of Education of Brazil, Brasília, Distrito Federal, Brazil; Duke University, United States of America

## Abstract

A new device was designed to generate a localized mechanical vibration of flexible gels where human umbilical vein endothelial cells (HUVECs) were cultured to mechanically stimulate these cells at subcellular locations. A Fluorescence Resonance Energy Transfer (FRET)-based calcium biosensor (an improved Cameleon) was used to monitor the spatiotemporal distribution of intracellular calcium concentrations in the cells upon this mechanical stimulation. A clear increase in intracellular calcium concentrations over the whole cell body (global) can be observed in the majority of cells under mechanical stimulation. The chelation of extracellular calcium with EGTA or the blockage of stretch-activated calcium channels on the plasma membrane with streptomycin or gadolinium chloride significantly inhibited the calcium responses upon mechanical stimulation. Thapsigargin, an endoplasmic reticulum (ER) calcium pump inhibitor, or U73122, a phospholipase C (PLC) inhibitor, resulted in mainly local calcium responses occurring at regions close to the stimulation site. The disruption of actin filaments with cytochalasin D or inhibition of actomyosin contractility with ML-7 also inhibited the global calcium responses. Therefore, the global calcium response in HUVEC depends on the influx of calcium through membrane stretch-activated channels, followed by the release of inositol trisphosphate (IP3) via PLC activation to trigger the ER calcium release. Our newly developed mechanical stimulation device can also provide a powerful tool for the study of molecular mechanism by which cells perceive the mechanical cues at subcellular levels.

## Introduction

Mechanical cues, such as substrate properties and mechanical forces, can affect a wide variety of cell behaviors and diseases [1]. For example, the substrate stiffness where cells are cultured can be determinant to the differentiation of stem cells, embryogenesis [2], and cell migration [3]. Different patterns of fluid shear stress may also be related to the development of atherosclerosis [4]. However, it remains unclear how cells perceive mechanical forces and correspondingly coordinate intracellular molecular signals.

Different techniques have emerged to allow the analysis of influence of mechanical forces in cellular biochemistry. For example, by adjusting the suction forces, patch-clamp techniques can induce the conformational changes of ion channels and the alteration of their properties [5]. Force can also be applied to cells through fluid shear stress in a flow chamber [4], [6] or by stretching a flexible substrate where the cells are plated on, such as a silicone membrane [7]. Another way to deliver mechanical forces is through laser tweezers [8], where a laser trap system is able to apply and measure forces on a cell-bound bead in the order of pN. Beads previously attached to the cell membrane can also be used to apply force from a glass probe [9] or simply from a magnetic field, if the beads are magnetized [10], [11]. Glass probes were also shown to touch the cells and evoke calcium signaling [12].

The intracellular calcium concentration has been shown to play crucial roles in a variety of physiological consequences and is sensitive to mechanical cues. In fact, shear stress can cause the rise of intracellular calcium concentration, which is involved in many signaling pathways, like the production of nitric oxide [6]. When actin stress fibers were directly stretched by optical tweezers or when fibronectin-coated beads previously attached to the apical surface of the cell were moved, stretch-activated calcium channels can be opened to trigger the alteration of intracellular calcium signals [9]. An increase in intracellular calcium concentration can also be observed to propagate among astroglial cells after mechanical stimulation by a glass probe touch [12]. Other reports indicate that the local deformation of a cell substrate caused an increase in intracellular calcium concentration of NIH3T3 cells, accompanied by changes in traction forces and cell orientation [13]. Calcium can also be involved in actomyosin contractility to regulate the tension in stress fibers. In fact, calcium was shown to change mechanical properties of cells, such as the stiffness of apical surfaces in HUVECs [10].

To visualize intracellular calcium concentration in real time with high spatiotemporal resolution, one could use calcium dyes, such as Fura-2 [14]. Another way is to use genetically encoded molecular biosensors. For example, Cameleon, a fluorescence resonance energy transfer (FRET) biosensor based on the interacting pair Calmodulin and M13, can allow the detection of intracellular calcium concentration with high precision [15], [16]. These biosensors have the advantage over calcium dyes of targeting subcellular regions or compartments instead of being diffused in the cytoplasm as a typical dye does [17]. We have previously discovered a FRET pair, ECFP and YPet, which allows a high sensitivity for the detection of a variety of molecular activities [11], [17]. The FRET pair in Cameleon was also replaced by ECFP and YPet and applied throughout this work to monitor the mechanical-force-induced calcium signaling.

In this work, a mechanical stimulation was applied to a human umbilical vein endothelial cell (HUVEC) by vibrating a probe positioned close to the cell on an elastic gel. The vibration of the gel caused deformations over a limited distance from the probe and the cell received only a local stimulation at subcellular levels. Cells were shown to have a significant calcium response upon this mechanical stimulation, which is dependent on the coordinated calcium influx across the plasma membrane as well as the calcium release from ER.

## Methods

### Cell culture

Human umbilical vein endothelial cells (HUVECs), pooled from multiple donors, were generously provided by Prof. Shu Chien's laboratory (University of California, San Diego). The cells were cultured in 60×15 mm cell culture dishes (Corning) in 5% CO_2_ at 37°C and passed when achieved confluency. Growth medium was changed every other day or when the confluency reached 70% or more. The growth medium was composed by 25% Endothelial Cell Growth Medium (Cell Applications, 211–500), 20% Fetal Bovine Serum (Atlanta Biologicals, S11050H), 52% Medium 199 (Gibco, 11150), 1% Penicillin-Streptomycin (Gibco, 15140), 1% L-Glutamine (Gibco, 25030) and 1% Sodium Pyruvate (Gibco, 11360).

### Genetically encoded FRET biosensor

A genetically encoded FRET biosensor based on ECFP and YPet was used to monitor the intracellular calcium concentration as previously described [11], [15]–[17]. The method of choice for delivering the DNA into the cell was the adenovirus infection (Adeno-X Expression System 1, Clontech), in which the biosensor plasmid was incorporated. Between 16 and 18 hr before the infection, the cells were passed from a confluent dish into a 35×10 mm glass-bottom dish. The cells were then infected with the adenovirus carrying the FRET biosensor and the infected medium was changed to normal growth medium after 4 hr of incubation. In the next day, the infected cells were passed to polyacrylamide gel dishes at the count of around 1,000 cells/dish. These gel dishes were ready for the vibration experiments about 20 hr later.

### Polyacrylamide gel preparation

The polyacrylamide gel dishes were prepared according to a well-established protocol [3] with the following reagents: Acrylamide at 8% (40% solution stock, Bio-Rad, 161-0140), Bis at 0.13% (2% solution stock, Bio-Rad, 161-0142), TEMED at 1∶2000 (Bio-Rad, 161-0801) and 10% w/v Amonium Persulfate at 1∶200 (Bio-Rad, 161-0700) in 10 mM HEPES buffer (Sigma, H4034). The stiffness of the gels was 20 kPa approximately [18]. The gels were cast on 35×10 mm glass-bottom dishes using a 12 mm round cover glass (Fisher, 12-545-80) to shape the droplet of gel solution. The volume of the gel solution droplet was calculated to allow an average thickness of 70 µm. To obtain the gel with beads, a second layer of polyacrylamide with embedded beads was prepared on top of the clear gel layer in a similar fashion [19], resulting in almost all of the beads in a single layer and at the same focal plane when observed through a 40×/0.75 objective. In brief, the solution for the polyacrylamide was mixed with a 1 µm-bead suspension (Invitrogen, F-8821) at 1∶250. A small amount of this gel solution containing beads was applied on top of the first gel layer and pressed by a cover glass on top to create a very thin polymerized gel layer embedded with beads (around <1 µm). Measurements of the thickness of these 2 combined layers with a calibrated micrometer showed no noticeable difference in thickness when compared to that of the first gel layer alone. The beads were also confirmed to localize at the same focal plane when observed through the microscope. To allow cell attachment, gel surfaces were then coated with the UV-activatable cross-linker Sulfo-SANPAH (Thermo Scientific, 22589). After the activation, the gel is coated with bovine fibronectin (Sigma, F1141) at 2 µg/cm^2^ and maintained in the cell culture incubator overnight. The gels were rinsed with Phosphate Buffered Saline (PBS) and incubated in growth medium for 15 min before the cells were passed and seeded on them.

### Stimulation equipment

**Figure 1:**
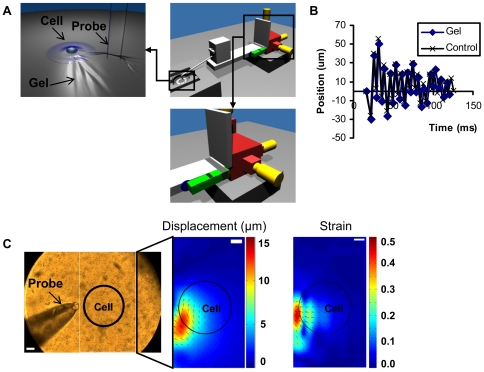
Mechanical stimulation equipment and vibration characterization. (A) Diagrams of vibration stimulation equipment with different components: XYZ stage (red), micrometers (yellow), mass-spring system (green) that generates the vibration and spacer (blue) used to trigger the vibration. The left image shows the cell stimulation caused by the vibration of the probe tip inserted in the flexible substrate gel. The right image shows the vibration generation part. (B) The time course of probe tip displacement when positioned in gel (Gel) or out of the gel (Control). (C) The left image shows the probe and gel substrate with 1 µm beads embedded. The right images show a typical displacement (in µm) and strain maps, with the cold and hot color representing the small and large displacement/strain, respectively. In both colormaps, the vectors represent the displacement directions on the substrate. The black circle represents the typical position of a cell (∼125 µm of diameter) during stimulation experiments. The bars represent 25 µm.

The stimulation equipment was designed to insert the probe tip in the substrate gel and cause a vibration of the gel where cells were seeded (Figure 1A, upper left panel). The probe tip consists of a glass capillary prepared by a micropipette puller and had a diameter of approximately 50 µm. The probe tip was mounted on a small aluminum rod attached to a small XYZ stage (Newport MT-XYZ model), which serves as a connector to an aluminum arm mounted on a larger XYZ linear stage (Newport 461 Series) (Figure 1A, large XYZ stage in red). The tip was precisely positioned by the larger XYZ linear stage 13 µm away from the cell edge and 25 µm deep penetrating into the substrate. Once positioned, the vibration mechanism was activated, locally vibrating the substrate and stimulating the cell. The vibration was dependent on an aluminum block clamped to the aluminum arm via a sheet of spring steel. The vibration was triggered by removing a spacer between the aluminum block and the arm (Figure 1A, aluminum block in green, spacer in blue). The resultant collision between the block and arm initiated a vibration, which was propagated through the structure and transmitted to the gel substrate by the small aluminum rod and probe tip (Video S1).

To keep the cell environment stable during imaging and vibration stimulation, a chamber was designed to allow the access of the probe to the cell dish as well as the constant entry of pre-mixed and humidified 5% CO_2_ (along with 10% O_2_ and 85% N_2_). A controlled heater (Nevtek ASI 400) was connected to maintain the temperature around 37°C throughout the experiment in the chamber.

### Imaging

A Zeiss fluorescence microscope equipped with an oil-immersed 40×/1.3 objective was controlled by a computer through the software MetaFluor 6.3r7 (Molecular Devices) to obtain the live cell images of the biosensor FRET signals. The xenon arc lamp can excite the donor fluorophore ECFP by using a 420/20 nm filter. 475/40 nm and 535/25 nm filters were used to observe the emissions from the donor (ECFP) and acceptor (YPet), respectively. For all samples, the cells were imaged for a few minutes to record the basal FRET ratio. The imaging was then paused to place the probe tip in position and resumed immediately after the mechanical stimulation was triggered.

For the vibration characterization, a fast camera (Vision Research Phantom v9.1) with 1,000 frames/second capacity was coupled to a phase contrast microscope with a 40×/0.75 objective to image the trajectory of the probe tip and the displacement caused on the flexible substrate gel containing beads. An extra halogen light was employed to obtain enough illumination for the high frame rates. These experiments were conducted at the Imaging Technology Group at the Beckman Institute for Advanced Science and Technology, University of Illinois at Urbana-Champaign.

### Postexperimental Imaging Analysis

Live cell images of the biosensors were captured and analyzed by MetaFluor 6.3r7. In the screenshot captured by the camera, the probe tip was positioned below the monitored cell and vibrated in the horizontal direction. The cell was divided vertically in three regions with equal height: (1) closer to the probe, (2) middle of the cell, and (3) farther from the probe. The FRET ratio was calculated from each region by dividing the average intensity of YPet by that of ECFP. The classification of response in global or local depended on the ratios of FRET signal over the regions closer and farther from the probe. The response was deemed global if the stimulated FRET increase on the farthest region away from the probe was at least half of that in the closest region. Otherwise, the response was deemed local. This criterion is systematic and robust, which allowed the comparison between different groups with statistical means and significance. If the ratio change upon stimulation remained within 10% of the basal level, it was considered as non-responsive. A region not covered by the cells and away from the probe tip was selected to assess the background signal and subtracted from each image.

To characterize the vibration, the images from the fast camera were analyzed using an ImageJ (available at http://rsb.info.nih.gov/ij; developed by Wayne Rasband, National Institutes of Health, Bethesda, MD) plugin, bUnwarpJ [20]. This plugin was used to compare the position of the beads between two frames: a frame displaced by the probe motion and a reference frame with the probe merely positioned but not moving. Matlab (MathWorks) was applied to reconstruct the displacement map obtained from bUnwarpJ, plotting it as a colormap combined with the vectors for a convenient and clear comprehension of the data. A colormap for the strain at each point was also calculated and plotted, with vectors denoting the displacement directions. The total strain *E* was calculated as:
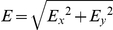
where E_x_ and E_y_ are the strain in x and y axis.
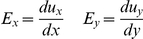
where u_x_ and u_y_ are the displacements in x and y axis, with each derivative numerically calculated by finite differences (2-point estimation for the boundaries, 3-point estimation for interior points).

### Statistical analysis

The classification of the response in global, local, or non-responsive can be treated as a multinomial distribution, with probabilities *p_g_*, *p_l_* and *p_n_*, respectively. For each condition, the probability distributions were estimated as following:
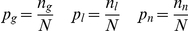
where *n_g_* is the number of global responses, *n_l_* is the number of local responses, *n_n_* is the number of non-responsive samples and *N* is the total number of samples for this particular condition.

The standard deviation of these probabilities obtained through this method can be estimated as:
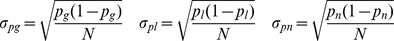



The p-value was calculated with a Fisher's exact test for the analysis of a contingency table to verify independence between the control and each of the drug treatments. The contingency table contained the number of samples that showed global, local or no response for each of the conditions.

### Reagents and inhibitors

EGTA (Calbiochem, 324625) was applied at 5 mM for 30 min. Gadolinium chloride (Sigma, 439770) was applied to cells at 3 µM for pretreatment of 20 min. Streptomycin (Sigma, S9137) was applied to cells at 200 µM for pretreatment of 10 min. Thapsigargin (Sigma, T9033) was applied at 1 µM for 45 min. U73122 (Sigma, U6756) was applied at 5 µM for 10 min. ML-7 (Sigma, I2764) was applied at 10 µM for 1 hr. Cytochalasin D (Sigma, C8273) was applied at 0.2 µM for 1 hr.

## Results

### The characterization of mechanical stimulation

The vibration frequency and magnitude of the probe tip generated by the mechanical stimulation equipment (Figure 1A) were determined while inserted inside the gel substrate or positioned out of the gel. For both cases, the vibration frequency was around 140 Hz (period of oscillations around 7 ms) and the probe tip displacement was around 70 µm in amplitude (Figure 1B). Thus, the interaction with the flexible gel substrate did not affect the probe tip displacement. The displacement distribution map of the gel substrate revealed that the maximum displacement (around 13∼14 µm) occurred at a position approximately 13 µm away from the probe edge, where the cells were positioned (Figure 1C, Video S2 and Video S3). Further results revealed that the maximum strain at the cell position was around 30%∼40%. Both displacement and strain were larger at regions closer to the stimulation site and became negligible at the farthest cell edge away from the probe (Figure 1C, Video S3 and Video S4).

### The mechanical stimulation of HUVECs

**Figure 2:**
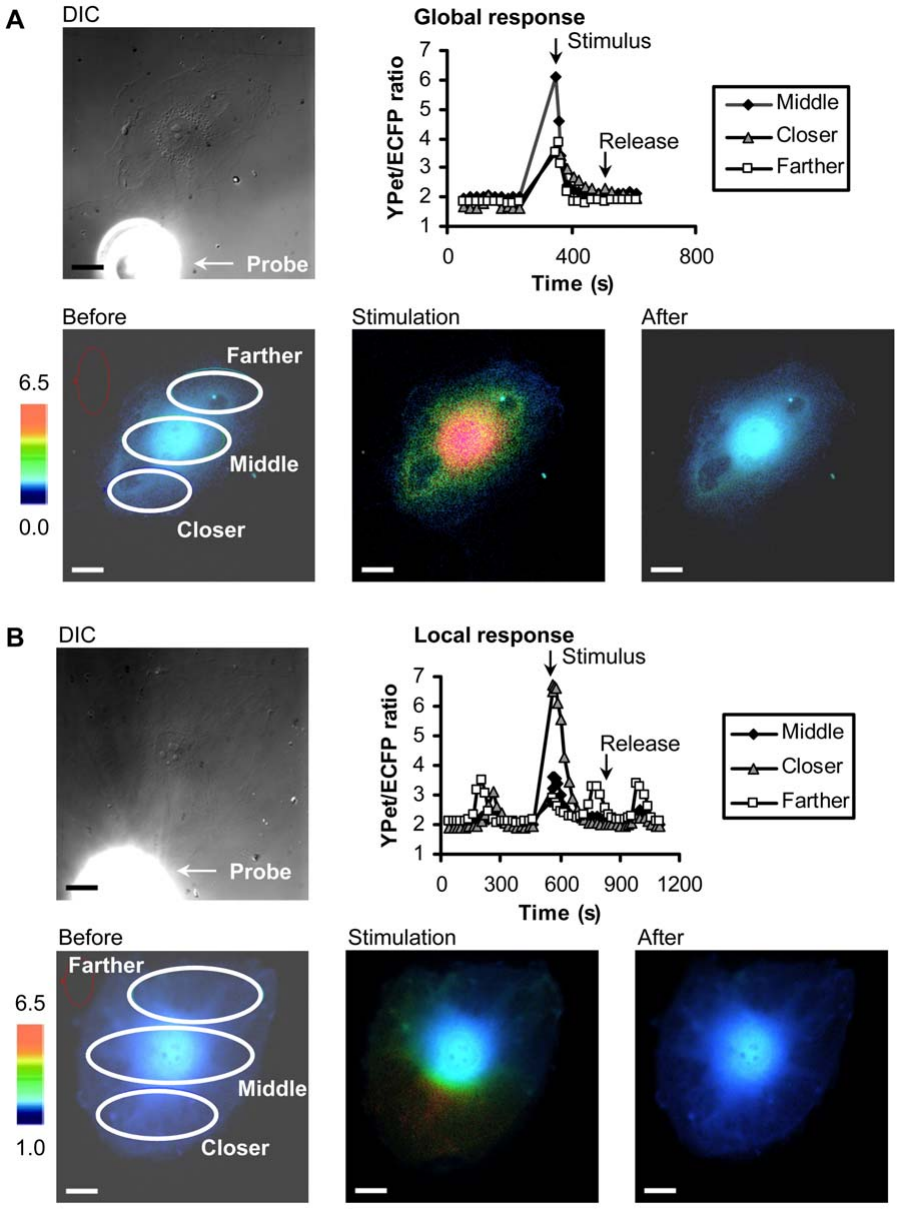
Types of cellular calcium responses upon mechanical vibration stimulation. (A) Global response represents a rise in intracellular calcium concentration occurring all over the cell body. (B) Local response represents a rise in intracellular calcium concentration occurring mostly at a region close to the stimulation site. The DIC images show the probe and a nearby cell. The time courses were shown to represent the intracellular calcium concentration in regions close (closer), median (middle), or far away from stimulation site. The color images represent the fluorescence emission ratio of YPet/ECFP from the biosensor before stimulation, immediately after stimulation, and after the calcium concentration re-stabilized. The bars represent 25 µm.

When this mechanical stimulation was applied on HUVECs (“Control” in experiments), it was possible to observe two different kinds of calcium signaling responses: global responses, in 80% of the samples, or local responses, in 20% of the samples. The global calcium response was characterized by a FRET response in the farther region of the cell away from the probe being more than half of that in the closer region (Figure 2A, Video S5), whereas the local response had calcium rise restricted to cell areas close to the probe tip but with little or no response at the distal part of the cell body (Figure 2B, Video S6).

### The dependence of extracellular calcium

Since intracellular calcium concentration can be affected by the calcium influx across the plasma membrane and the calcium release from intracellular calcium stores, we first assessed the effect of extracellular calcium in the calcium response upon this mechanical stimulation. Since calcium influx can occur through membrane stretch-activated channels as previously demonstrated [9], two approaches were applied for this purpose: (1) eliminating extracellular calcium and (2) blocking ion channels on the plasma membrane.

**Figure 3:**
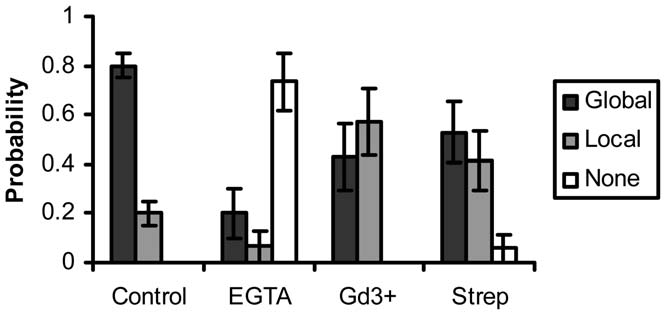
Extracellular calcium influx across the plasma membrane channels mediates the calcium response upon mechanical stimulation. The bar graphs represent the percentage of cells with global, local, or none responses under different treatment as indicated, with the error bars representing the respective standard deviations. EGTA (extracellular calcium chelator): *p* = 1×10^−10^, Gadolinium (Gd3+, stretch-activated calcium channel blocker): *p* = 0.007, Streptomycin (Strep, stretch-activated calcium channel blocker): *p* = 0.02. The statistical difference from the control group is determined by *p*<0.05. All results were significantly different from control.

After chelating the extracellular calcium from the growth medium with EGTA [16], [21]–[23], the response was abolished from the majority of the cells (Figure 3, “EGTA”). Gadolinium chloride (Gd3+) treatment to block stretch-activated membrane calcium channels [9], [24] resulted in local responses in the majority of the cells (Figure 3, “Gd3+”). Streptomycin (Strep) treatment to block stretch-activated membrane calcium channels [24]–[30] also reduced the occurrence of global responses significantly (Figure 3, “Strep”). Based on the statistical analysis, all reagents affected significantly the calcium response upon mechanical stimulation (EGTA: *p* = 1×10^−10^, Gd3+: *p* = 0.007, Strep: *p* = 0.02). These data suggest that the global calcium responses are dependent on the influx of extracellular calcium through the plasma membrane via stretch-activated channels.

### The dependence of intracellular calcium stores (Endoplasmic Reticulum – ER)

In global responses, significant calcium signaling was observed more than 50 µm away from the stimulation site, where there were minimal mechanical stretch applied. This suggests that the calcium may be released from intracellular stores to cause the global responses.

**Figure 4:**
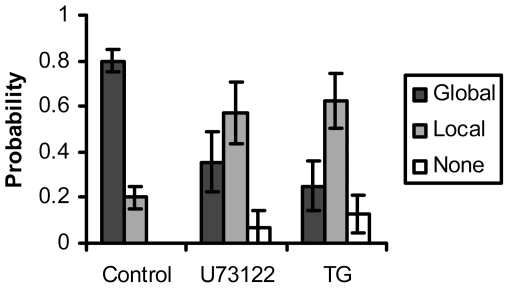
PLC and ER calcium mediate the calcium response upon mechanical stimulation. The bar graphs represent the percentage of cells with global, local or none responses under different treatment as indicated, with the error bars representing the respective standard deviations. Thapsigargin (TG, ER calcium pump inhibitor): *p* = 1×10^−5^, U73122 (PLC inhibitor): *p* = 7×10^−4^. The statistical difference from the control group is determined by *p*<0.05. All results were significantly different from control.

Thapsigargin, which inhibits endoplasmic reticulum calcium pump and depletes the intracellular calcium stores [31], resulted in a majority of local responses (Figure 4, “TG”). A phospholipase C (PLC) inhibitor U73122 [32], [33], which blocks the release of inositol trisphosphate (IP3), also showed a majority of local responses (Figure 4, “U73122”). The effect from either Thapsigargin or U73122 was statistically significant from the control cells without treatment (TG: *p* = 1×10^−5^, U73122: *p* = 7×10^−4^). Therefore, the global calcium responses upon mechanical stimulation also depend on the release of calcium from intracellular calcium stores through IP3 signaling (Figure 4).

### The dependence on actomyosin contractility

**Figure 5:**
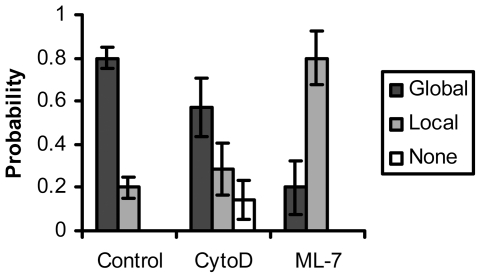
Actomyosin contractility and actin cytoskeleton mediate the calcium response upon mechanical stimulation. The bar graphs represent the percentage of cells with global, local or none responses under different treatment as indicated, with the error bars representing the respective standard deviations. ML-7 (myosinlight chain kinase inhibitor): *p* = 3×10^−4^, Cytochalasin D (CytoD, f-actin polymerization inhibitor): *p* = 0.01. The statistical difference from the control group is determined by *p*<0.05. All results were significantly different from control.

Cytoskeleton and its related actomyosin contractility play an important role in transmitting forces throughout the cell [11] and in activating stretch-activated channels [9]. We have hence investigated the role of actomyosin contractility by applying ML-7 to inhibit the function of the myosin light chain kinase and hence actomyosin contractility [34]. ML-7 treatment caused a majority of local responses (Figure 5, “ML-7”). The disruption of F-actin with cytochalasin D (CytoD) [9], [35] at 0.2 µM also inhibited the global calcium responses upon mechanical stimulation (Figure 5, “CytoD”). The calcium responses of cells treated by either ML-7 or CytoD were significantly inhibited from that of control cells (ML-7: *p* = 3×10^−4^, CytoD: *p* = 0.01). Thus, the full calcium responses upon mechanical stimulation also depend on the actomyosin contractility and F-actin integrity.

## Discussion

**Figure 6:**
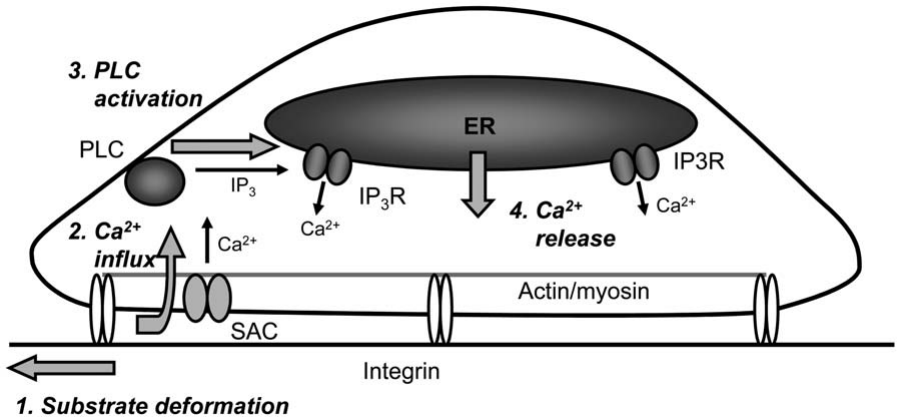
Schematic diagram depicting the hypothesized mechanism of calcium response upon mechanical vibration stimulation. The substrate gel deformation causes a calcium influx through the membrane stretch-activated channels (SAC), activating phospholipase C (PLC). PLC then produces IP3 and calcium is subsequently release from intracellular stores (ER) due to IP3 signaling (through IP3 receptors, IP3R).

In this work, we have developed a new probe device to mechanically stimulate cells at precisely controlled subcellular locations without the direct contact with cell membrane. The results indicate that a mechanical vibration induced by the device in the substrate gel where cells are seeded can mainly cause global calcium responses of the cells. This global response was initiated by the influx of calcium across the stretch-activated channels in the plasma membrane. The subsequent production of IP3 via PLC activation triggers the calcium release from ER to cause a global intracellular calcium fluctuation over the whole cell body. This global calcium response was also shown to depend on actomyosin contractility and F-actin integrity, probably controlling the membrane stretch-activated channels. This whole mechanistic scenario is summarized in the diagram of Figure 6.

Although a simple stretch of the substrate gel with a micro-needle has been nicely employed to mechanically stimulate cells by Krishnan et al [36], the high temporal frequency and repeated pattern of the vibration stimulation of our system should be able to elicit a stronger and robust signal. Indeed, when the stimulation was manually applied while maintaining the same magnitude of stretch, no calcium response was observed in the cells (data not shown). This is consistent with a previous report that mechanical stimulation with a faster transient acceleration rate can trigger stronger cellular responses in neuronal cells [37]. Higher temporal gradients was also more effective than spatial gradients when stimulating endothelial cells with shear stress [4]. The strain and strain rate imposed on the substrate in this study were similar to those used for other cell studies involving mechanical stretch and calcium signaling, both towards cell injury: one involving rat primary hippocampal neuronal cells with strains up to 28% in a cycle as short as 70 ms [37]; another involving human pulmonary microvascular endothelial cells with applied uniaxial strains up to 30% for 3 sec [38]. Thus, the strain and strain rate were larger at regions closer to the probe, which showed the localized nature of the stimulation delivered and was consistent to the region of stronger intracellular calcium rise in local responses. This localized stimulation was essential to study the details of the calcium signaling propagation.

Hence, the main motivation of our device development was to build a system capable of delivering a local and robust mechanical stimulation with highly integrated spatiotemporal characteristics so that we can investigate the fundamental mechanism of mechanotransduction at subcellular levels. Another advantage of this technique is that it is less invasive to the innate cellular functions since there is no direct contact between the stimulating probe and the cell body, avoiding the possible membrane penetration by the probe or the integrin/cytoskeleton alteration and clustering around the adhesion beads before mechanical stimulation [10]. Therefore, our novel system can also allow a non-invasive mechanical stimulation at subcellular levels. Even though the present work was focused on calcium signaling, it is important to notice that the benefits of using this stimulation device can be extended to other signaling pathways as well. Indeed, the activation of signaling events other than the intracellular calcium may also be sensitive to temporal gradients of mechanical stimulation, such as ERK1/2 [4].

The extracellular calcium and its influx across the plasma membrane is apparently the trigger of the whole calcium signaling cascade as the chelation of extracellular calcium by EGTA or the blockage of stretch-activated channels on the plasma membrane by streptomycin and gadolinium chloride drastically inhibited the calcium response to mechanical stimulation. The activation of these stretch-activated channels may be mediated by actin cytoskeleton and actomyosin contractility as ML-7 and CytoD both inhibited the mechanical-stimulation-induced calcium response, which is also consistent with previous reports that the activation of stretch-activated channels are mediated by the actin filaments in HUVECs [9]. It is interesting that the inhibitory effect of CytoD was different from that of ML-7. It is possible that the activation of stretch-activated channels is affected mostly by the cortical actin network and the CytoD treatment may have a less inhibitory effect on short cortical actin networks than on long stress fibers. Blebbistatin should have similar effect as ML-7. However, Blebbistatin was not employed in this study since it introduces intensive background fluorescence which interferes with the wavelengths for the biosensor imaging.

The PLC/IP3 pathway and ER calcium release are clearly involved in the mechanically induced calcium response as the inhibition of PLCs by U73122 and the depletion of ER calcium by Thapsigargin inhibited the response. PLC-δ is usually inactive at basal calcium concentrations and activated when the intracellular calcium concentration rises [39]. Other proteins (such as RhoA and transglutaminase) can also act as regulators to lower the required activation concentration of calcium [40], [41]. Thus, it is possible for the rise of the local calcium concentration due to the mechanical stimulated membrane channel opening to activate PLC-δ, triggering the IP3 signaling pathway and ER calcium release to yield a global response.

## Supporting Information

Video S1 **Animation of the vibration equipment and its stimulation on a cell.** Animation showing the vibration stimulation equipment with different components: XYZ stage (red), micrometers (yellow), mass-spring system (green) that generates the vibration and spacer (blue) used to trigger the vibration. An illustration of the stimulation on a cell seeded on an elastic gel dish is also presented.
(AVI)

Video S2 **Polyacrylamide substrate deformation during vibration stimulation.** Video taken with a high-speed camera, at 1,000 frames per second. The polyacrylamide gel under deformation has 1 µm beads embedded close to the surface. The black circle represents the typical position of a cell (∼125 µm of diameter) during stimulation experiments.
(AVI)

Video S3 **Polyacrylamide substrate deformation plotted as a colormap/vectormap.** The deformation observed with the high-speed camera was utilized to calculate the spatial colormap of the displacement with vectors at different locations indicating the direction and magnitude at the local positions. The black circle represents the typical position of a cell (∼125 µm of diameter) during stimulation experiments. The bar represents 25 µm.
(AVI)

Video S4 **Polyacrylamide substrate strain plotted as a colormap.** The strain, calculated from the deformation observed with the high-speed camera, was plotted as a spatial colormap, with vectors representing displacement directions. The black circle represents the typical position of a cell (∼125 µm of diameter) during stimulation experiments. The bar represents 25 µm.
(AVI)

Video S5 **Example of global calcium response upon mechanical stimulation.** Video of the colormap in a cell representing the intracellular calcium concentration measured by the fluorescence emission ratio of YPet/ECFP from the calcium biosensor before and after the mechanical stimulation. This particular cell displayed a global calcium response. The scale bar represents 25 µm.
(AVI)

Video S6 **Example of local calcium response upon mechanical vibration stimulation.** Video of a colormap representing the fluorescence emission ratio of YPet/ECFP from the biosensor before and after stimulation, with a cell displaying a local calcium response. The bar represents 25 µm.
(AVI)
